# Random Forest Approach in Modeling the Flow Stress of 304 Stainless Steel during Deformation at 700 °C–900 °C

**DOI:** 10.3390/ma14071812

**Published:** 2021-04-06

**Authors:** Shin-Hyung Song

**Affiliations:** Department of Smart Automobile, Soonchunhyang University, Asan-si 31538, Korea; neuro2@sch.ac.kr; Tel.: +81-41-530-4987

**Keywords:** 304 stainless, Arrhenius equation, flow stress, random forest

## Abstract

The alloy 304 stainless steel is used in a wide variety of industrial applications. It is frequently applied in tough environments, such as those involving high temperatures, low temperatures, and corrosive environments. Hence, research on the flow stress behavior of the alloy during deformation under tough environments is critically important to achieving the maximum effectiveness in the application of the alloy. This research presents a study on the flow stress of 304 stainless steel during hot deformation at the temperatures of 700 °C–900 °C under the strain rates ranging from 0.0002/s–0.02/s. For this study, hot tensile experiments are conducted, and the flow stress variations of the alloy are studied with respect to the variations in the strain rate and temperature. Next, the stress behavior was modeled by the traditional Arrhenius-type constitutive equation and random forest algorithm. Then, the flow stresses predicted by different methods were studied by comparing errors. The results showed that the flow stress was modeled more accurately by the random forest algorithm.

## 1. Introduction

The alloy 304 austenitic stainless steel has good corrosion resistance, as well as robust mechanical properties at high temperatures and aggressive environments [1,2,3]. Due to its superior properties, 304 stainless steel has been used in a variety of fields, including marine service, power plant, the nuclear sector, oil industries, etc. [1,2,3,4]. Correspondingly, the deformation characteristics and mechanical properties of 304 stainless and other austenitic stainless steel under various environments have represented one of the major research topics in the field. The deformation characteristics of austenitic stainless steel are complex, and the characteristics significantly depend on the temperature and strain rates.

To date, many studies have examined the deformation of austenitic stainless steel and modeled the flow stress [5,6,7,8,9,10]. Haj et al. [5] conducted hot compression experiments for AISI 321 austenitic stainless steel under temperatures ranging from 950 °C–1000 °C and strain rates ranging from 0.01/s–1/s. The experimental study found that the flow stress variation is significantly influenced by the deformation temperatures and strain rates. The flow stress of the AISI 321 austenitic stainless steel was modeled by a constitutive equation based on the Zener-Hollomon theory. Kotkunde et al. [6] carried out tensile experiments for austenitic stainless steel 316 at temperatures ranging from 150 °C–600 °C and strain rates ranging from 0.0001/s–0.01/s. In the study, the flow stress behavior of stainless steel 316 was modeled by the modified Fields-Backofen equation and the KHL (Khan-Huang-Liang) equation. The research showed that the KHL model is superior to the other model. Babu et al. [7] performed hot deformation tests for super austenitic stainless steel at temperatures ranging from 1173K–1423K and strain rates ranging from 0.001/s–10/s. In this study, the flow stress model of the stainless steel was developed based on the Zener-Hollomon parameters and regression methods. Pu et al. [8] studied the high temperature deformation of super austenitic stainless steel S32654 by conducting hot compression tests at temperatures ranging from 950 °C to 1250 °C and strain rates ranging from 0.001/s to 10/s. In this study, the flow stress of the stainless steel was modeled by Arrhenius-type constitutive equations. Cadoni et al. [9] carried out high-speed deformation tests for AISI304 stainless steel under strain rates ranging from 0.001/s–1000/s. In this research, the flow stress was modeled by a modified type of Johnson-Cook equation. Zhang et al. [10] performed the constitutive modeling for nitrogen-containing austenitic stainless steel 316LN by performing hot compression tests at temperatures ranging from 900 °C–1250 °C and strain rates ranging from 0.001/s–10/s. In that study, the flow stress of stainless steel 316LN was predicted using the Arrhenius-type equation.

Although modeling the flow stress by using the traditional constitutive equation has generally been successful, the approach has some drawbacks in predicting the accuracy. For example, the Arrhenius-type equation, which is known as one of the most popular and efficient equations for hot deformation study, has some drawbacks, such as the fact that, sometimes, the equation has limited accuracy and the fact that one needs to recalculate the new parameters in the equation when optimizing the equation with new data [11].

Due to the drawbacks of the traditional approach, many researchers have begun to conduct research on predicting the flow stress using a neural network approach [11,12,13,14,15,16]. Yan et al. [12] conducted hot compression tests for the Al−6.2Zn−0.70Mg−0.30Mn−0.17Zr alloy at temperatures ranging from 623K–773K and strain rates ranging from 0.01/s–20/s. The research used Arrhenius-type constitutive equations and an artificial neural network approach to predict the flow stress, and the results showed that the artificial neural network model predicted the flow stress more accurately and efficiently than the Arrhenius equations. Bobbili et al. [13] performed high-speed compression tests for high-strength armor steel at temperatures ranging from 500 °C–650 °C and strain rates ranging from 1000/s–5500/s. In that study, an artificial neural network model and the Johnson-Cook constitutive model were used to predict the flow stress, and the results showed that the artificial neural network model outperforms the Johnson-Cook model. Guo et al. [14] modeled the flow stress of the TC21 alloy during hot compression at strain rates and temperatures, respectively, ranging from 0.01/s–50/s and from 900 °C–1000 °C. The research showed that the developed model predicted the flow stress of the alloy with good accuracy. Quan et al. [15] also carried out hot compression tests for the AZ80 alloy at temperatures ranging from 523K–673K and strain rates ranging from 0.01/s–10/s. The flow stress was modeled using the Arrhenius-type equation and the BP-ANN (back propagation artificial neural network) model, and it was found that the BP-ANN predicted the flow stress with better accuracy than the Arrhenius-type equation. Han et al. [11] also performed a comparison between the Arrhenius-type model and the artificial neural network model in their ability to model the flow stress of as-cast 904L austenitic stainless steel, and they found that the neural network model is superior to the Arrhenius-type model. Zhu et al. [16] also used the ANN (artificial neural network) model to model the flow stress of as-cast titanium alloy during compression at 1000 °C–1150 °C and 0.01/s–10/s, and they compared the accuracy using the regression method. The research found that the ANN model outperforms the regression method.

Although the neural network approach of modeling the flow stress of hot deformation was quite successful, the neural network model still has some drawbacks in modeling the efficiency and computational cost. Specifically, the neural network model has to optimize the model by adjusting the process parameters, including the number of hidden layers, neurons in each layer, epochs, etc. Therefore, there is still a need to adopt a model with good modeling efficiency and prediction accuracy.

In the present study, a random forest algorithm [17] is adopted to model the flow stress of the hot deformation process. To this end, hot deformation testing for 304 stainless steel at various temperatures and strain rates was presented. The measured flow stress variation was modeled using traditional Arrhenius-type equations and a random forest algorithm. The presented model was also compared with other models in terms of the accuracy and other aspects of modeling.

## 2. Materials and Methods

**Figure 1:**
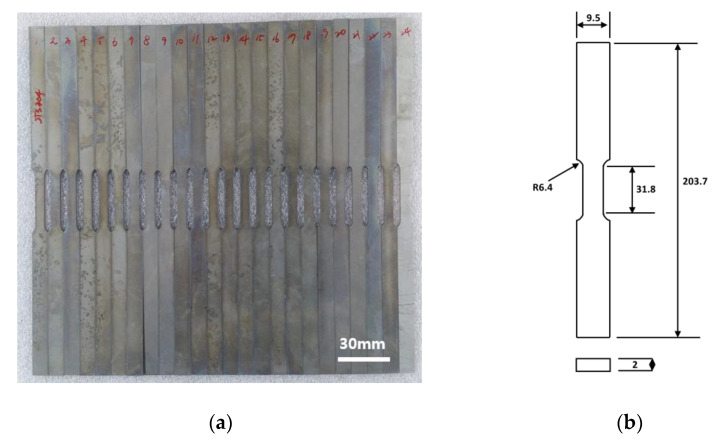
(**a**) Test specimens and (**b**) specimen geometry (dimensions are in mm).

For the high-temperature tensile test, specimens of 304 stainless steel were prepared. Wire-EDM (Electrical discharge machining) machining was used to machine the planar specimens. The dimensions of each specimen were about 203.7 mm in length, 9.5 mm in width, and 2 mm in thickness. The test specimens are heated in a furnace to high temperatures. Tensile tests were conducted in a testing machine of model MTS-810. Figure 1a shows the machined specimens, and Figure 1b shows the geometry of the test specimens in detail. In this research, the high-temperature tests were conducted at temperatures of 700 °C, 800 °C, and 900 °C and strain rates of 0.0002/s, 0.002/s, and 0.02/s.

### 2.1. Modeling by Arrhenius-Type Constitutive Equation

In this research, the Arrhenius-type equation with the Zener-Hollomon theory is used to model the flow stress of the high-temperature deformation of 304 stainless steel. When the function *F*(*σ*) is in the form shown below:

(1) $$F\left(\sigma \right)=\{\begin{array}{cc} \sigma^{n^{\prime }} & \alpha \sigma <0.8\\ \exp\left(\beta \sigma \right) & \alpha \sigma >1.2\\ {\left[\sinh\left(\alpha \sigma \right)\right]}^{n} & for\;all\;\alpha \sigma \end{array}$$

where $n^{\prime }$, α, β, and n denotes material constants, and the strain rate $\dot{\varepsilon }$ is described as

(2) $$\dot{\varepsilon }=AF\left(\sigma \right)\exp\left(\frac{-Q}{RT}\right)$$

where A is the material constant. The Zener-Hollomon parameter Z can be written as follows.

(3) $$Z=\dot{\varepsilon }\;\exp\left(\frac{Q}{RT}\right)$$

where T(K) is the temperature, $\dot{\varepsilon }\left(s^{-1}\right)$ denotes the strain rate of the deformation, $Q\left(J{\mathrm{mol}}^{-1}\right)$ is the activation energy, and $R\left(8.314\;J{\mathrm{mol}}^{-1}K^{-1}\right)$ denotes the gas constant.

Equation (4) is obtained after substituting the first Equation (1) into Equation (2) and taking the logarithms of both sides, as noted in Equation (4).

(4) $$\ln\sigma =\frac{1}{n^{\prime }}\ln\dot{\varepsilon }-\frac{1}{n^{\prime }}\ln B$$

From the $\ln\sigma -\;\ln\dot{\varepsilon }$ curves in the above equation, the material constant $n^{\prime }$ is obtained by calculating the slope of the curve. In the development of the Arrhenius equation described in this research, the necessary calculations to obtain n’ are done at strains ranging from 0.03–0.3, with an interval of 0.03.

After substituting the second part of Equation (1) into Equation (2) and taking the logarithms of the obtained equation, one can obtain the relationship shown in Equation (5). The material constant β is obtained by calculating the slopes of the $\sigma -\ln\dot{\varepsilon }$ curves.

(5) $$\sigma =\frac{1}{\beta }\ln\dot{\varepsilon }-\frac{1}{\beta }\ln C$$

The material constant α, which is defined as shown in Equation (6), is calculated using β and $n^{\prime }$.

(6) $$\alpha =\frac{\beta }{n^{\prime }}$$

β and α are also calculated at the strains of 0.03–0.3, with an interval of 0.03.

After substituting the third equation of (1) into (2) to take logarithms of the obtained equation, we obtain the equation shown in (7). The material constant n is obtained after calculating the slopes of the $\ln\dot{\varepsilon }-\ln[\sinh\left(\alpha \sigma \right)]$ curves. As with the other materials constants described above, n is also obtained for the same strain values as above.

(7) $$\ln[\sinh\left(\alpha \sigma \right)]=\frac{\ln\dot{\varepsilon }}{n}+\frac{Q}{nRT}-\frac{\ln A}{n}$$

The material constant Q is similarly calculated from the slope of the $\ln[\sinh\left(\alpha \sigma \right)]-\left(\frac{1}{T}\right)$ curve.

Finally, the material constant A is obtained by using the *y*-axis intercept of the lnZ−ln[sinh(ασ)] plot from Equation (8), which is derived from Equations (1) and (7).

(8) lnZ=lnA+nln[sinh(ασ)]

**Figure 2:**
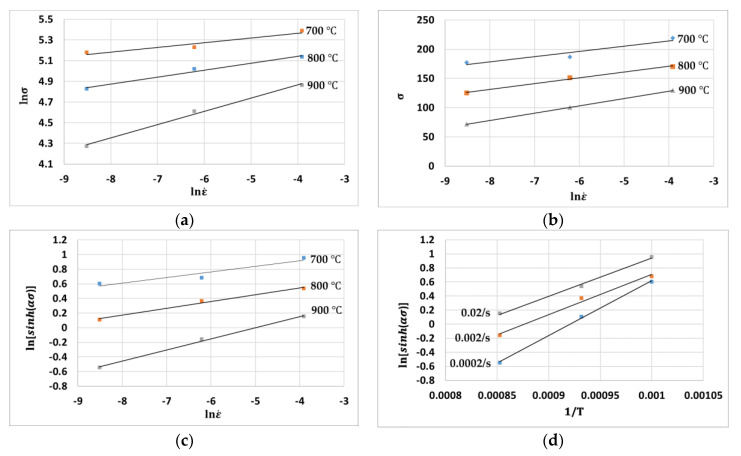
Plots of (**a**) ln$\sigma -\ln\dot{\varepsilon }$, (**b**) $\sigma -\ln\dot{\varepsilon }$, (**c**) $\ln\dot{\varepsilon }-\ln[\sinh\left(\alpha \sigma \right)]$, and (**d**) $\ln[\sinh\left(\alpha \sigma \right)]-\left(\frac{1}{T}\right)$.

Similar to the other material constant, Q and A are also obtained at various strain values ranging from 0.03–0.3 with an interval of 0.03. Figure 2 shows the plots of (a) ln$\sigma -\ln\dot{\varepsilon }$, (b) $\sigma -\ln\dot{\varepsilon },$ (c) $\ln\dot{\varepsilon }-\ln[\sinh\left(\alpha \sigma \right)]$, and (d) $\ln[\sinh\left(\alpha \sigma \right)]-\left(\frac{1}{T}\right)$ for the strain of 0.03.

Since the material constants A, $n^{\prime }$, α, β, and n are the functions of the strain, the constants n, α, Q, and A can be described as the 7th-order polynomial function, as in Equations (9)–(12). A regression analysis was conducted to determine the coefficients of the polynomials. Table 1 presents the list of the calculated coefficients of n, α, Q, and A. Table 2 presents the RMSE (root mean square error) of the material constants calculated by the polynomial functions.

(9) $$\alpha =\alpha_{7}\varepsilon^{7}+\alpha_{6}\varepsilon^{6}+\alpha_{5}\varepsilon^{5}+\alpha_{4}\varepsilon^{4}+\alpha_{3}\varepsilon^{3}+\alpha_{2}\varepsilon^{2}+\alpha_{1}\varepsilon^{1}+\alpha_{0}$$

(10) $$n=n_{7}\varepsilon^{7}+n_{6}\varepsilon^{6}+n_{5}\varepsilon^{5}+n_{4}\varepsilon^{4}+n_{3}\varepsilon^{3}+n_{2}\varepsilon^{2}+n_{1}\varepsilon^{1}+n_{0}$$

(11) $$Q=q_{7}\varepsilon^{7}+q_{6}\varepsilon^{6}+q_{5}\varepsilon^{5}+q_{4}\varepsilon^{4}+q_{3}\varepsilon^{3}+q_{2}\varepsilon^{2}+q_{1}\varepsilon^{1}+q_{0}$$

(12) $$A=a_{7}\varepsilon^{7}+a_{6}\varepsilon^{6}+a_{5}\varepsilon^{5}+a_{4}\varepsilon^{4}+a_{3}\varepsilon^{3}+a_{2}\varepsilon^{2}+a_{1}\varepsilon^{1}+a_{0}$$

**Figure 3:**
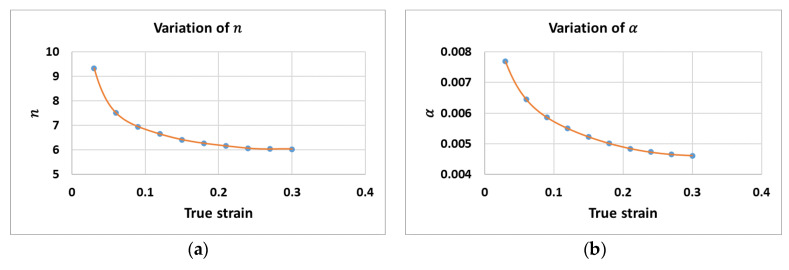
Variation of (**a**) n and (**b**) α.

**Figure 4:**
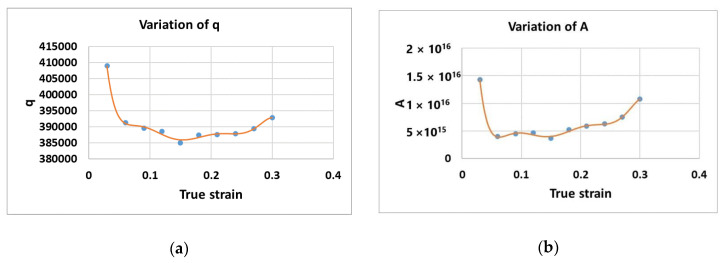
Variation of (**a**) q and (**b**) A.

Figure 3 shows the variations of (a) n and (b) α. Meanwhile, Figure 4a and Figure 4b present the variations of Q and A, respectively.

The final form of the equation describing the flow stress with the Zener-Hollomon parameter is obtained using Equations (1) and (2) after calculating all the material constants. The equation of stress is as shown below in Equation (13).

(13) $$\sigma =\frac{1}{\alpha }\ln\left[{\left(\frac{Z}{A}\right)}^{\frac{1}{n}}+{\left[{\left(\frac{Z}{A}\right)}^{\frac{2}{n}}+1\right]}^{\frac{1}{2}}\right]$$

### 2.2. Modeling by Random Forest Algorithm

In a random forest algorithm, an ensemble method of learning is performed by a forest structure of multiple decision trees. To understand the random forest algorithm in detail, it is worth mentioning the decision tree first. The decision tree algorithm splits the original dataset at each classifying point, called a node, with certain classifying attributes. Starting from the initial point called the root node (top node), the datasets repeat being split into smaller classes through continuous actions by different attributes at child nodes (sub-nodes). The decision tree algorithm is originally a splitting algorithm. However, the algorithm can be utilized as a regression algorithm when the target classes are numeric. To this end, this decision algorithm has been effectively adopted in many classifying and regression works. To obtain even greater effectiveness, a random forest algorithm is developed by utilizing multiple tree structures. Each decision tree becomes a predictor, and predictions from trees are used to choose the most popular prediction.

In this research, the python/scikit learn package was used to adopt a random forest algorithm in a regression problem. In the developed random forests model, the input attributes were the strain rate, temperature, and strain; the output attribute was stress. From each stress–strain curve, the strain range from 0.03–0.3 was chosen as the area of research, and 10 data points from each curve were chosen at strains ranging from 0.03–0.3, with a 0.03 interval. Therefore, 90 data points in total were chosen as the test set. In addition, 40 data points were randomly chosen from each curve (at the range of 0.03–0.3) to be the learning set, and hence, 360 data points in total were used as the learning set. Bootstrapping was used in this calculation to avoid overfitting in the regression. Table 3 lists the process parameters of the random forest algorithm used in this research.

## 3. Results

### 3.1. Experimental Results

**Figure 5:**
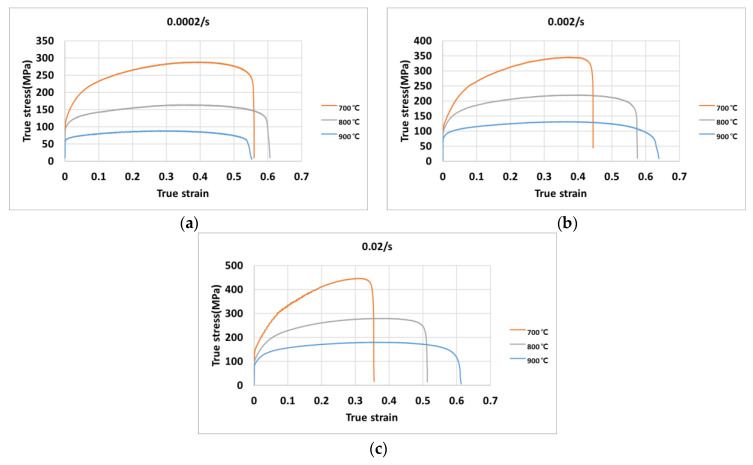
Stress–strain curves at the strain rates of (**a**) 0.0002/s, (**b**) 0.002/s, and (**c**) 0.02/s.

The experimental data was measured to calculate the true-stress and true-strain at the temperatures of 700 °C, 800 °C, and 900 °C under the strain rates of 0.0002/s, 0.002/s, and 0.02/s. The true-stress–true-strain curves are plotted for each temperature with different strain rates in Figure 5a–c. The results show that the flow stress behavior significantly depends on the variation of the strain rate and temperature. The stress increases when the strain rate increases, and it decreases when the temperature increases.

### 3.2. Arrhenius-Type Constitutive Equation

**Figure 6:**
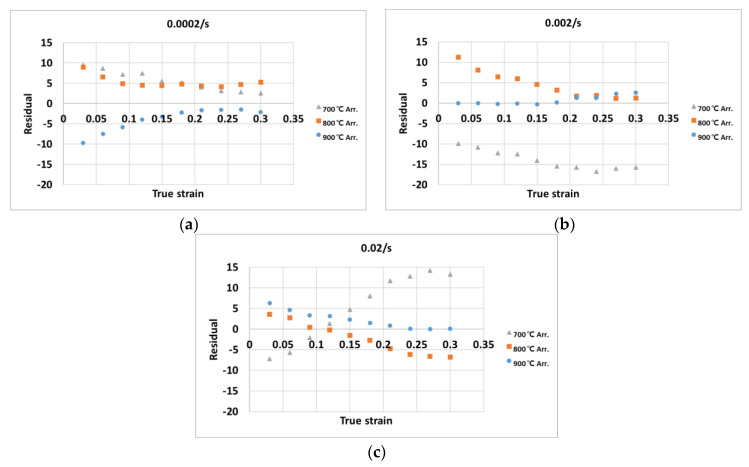
Residual plots of the calculated stress by the Arrhenius-type equation at (**a**) the strain rate 0.0002/s, (**b**) strain rate 0.002/s, (**c**) strain rate 0.02/s.

Figure 6 shows the residual plots of the stress values predicted by the Arrhenius-type equation at the strains of (a) 0.0002/s, (b) 0.002/s, and (3) 0.02/s. The flow stress predicted by the traditional constitutive equation is quite similar to the experimental values. However, at certain strain rates and temperatures, the error can be clearly seen (for example, at a strain rate of 0.002/s and a temperature of 700 °C), as the RMSE of the stress values predicted by the constitutive equation was 6.93.

### 3.3. Random Forest Algorithm

**Figure 7:**
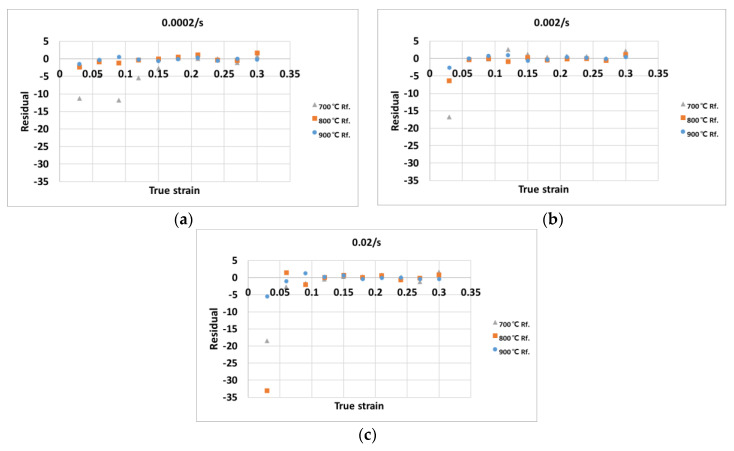
Residual plots of the predicted stress by the random forest algorithm at (**a**) the strain rate 0.0002/s, (**b**) strain rate 0.002/s, and (**c**) strain rate 0.02/s.

Figure 7 shows the residual plots of the stress values predicted by the random forest algorithm at the strains of (a) 0.0002/s, (b) 0.002/s, and (3) 0.02/s. The flow stress predicted by the random forest algorithm is very similar to the experimental values. Although the predicted values show an obvious error at certain temperatures and strain rates (for example, the temperatures of 700 °C and 800 °C and at the strain rate of 0.02/s), the RMSE of the predicted values was 4.93, which is better than the stresses predicted by the constitutive equation.

## 4. Discussion

**Figure 8:**
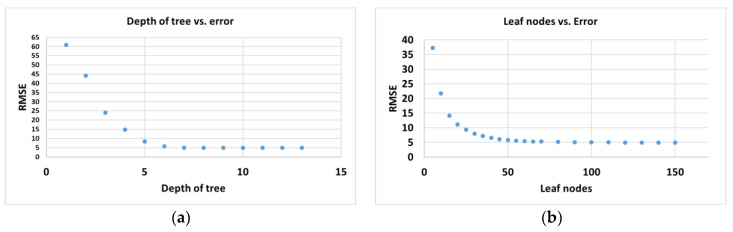
(**a**) RMSE vs. maximum depth of the tree and (**b**) RMSE vs. maximum leaf nodes.

An analysis of the influence of the maximum depth and leaf nodes of the tree structure of the random forest algorithm on the accuracy of the model was performed. Figure 8 shows the (a) RMSE versus the maximum depth of the tree and (b) RMSE versus the maximum leaf nodes.

In this research, developing the prediction model by the random forest approach could be done efficiently, and the developed model was computationally inexpensive. The advantages of the random forest algorithm could be noticeable when one considers other computationally expensive algorithms (e.g., neural networks.).

**Figure 9:**
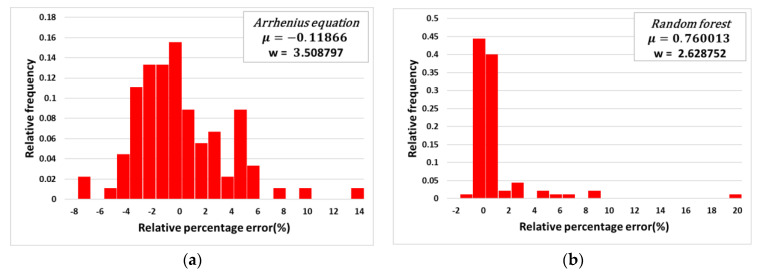
Plots of the relative percentage error by (**a**) the Arrhenius-type equation and (**b**) random forest algorithm.

The analysis of the relative percentage error was conducted for the flow stress values calculated by the Arrhenius constitutive equation and the random forest algorithm. Figure 9 shows the relative percentage errors versus the relative frequency for the (a) Arrhenius constitutive approach and (b) random forest approach. For each approach, the mean value of the relative percentage error, indicated as μ, was about −0.12 and 0.76, respectively. The standard deviation for the relative percentage error, indicated as w, was about 3.51 and 2.63, respectively. In other words, the absolute mean value of the relative percentage error for the random forest approach was larger than the error obtained by the Arrhenius constitutive equation. However, the standard deviation of the relative percentage error for the Arrhenius constitutive equation was larger than that for the random forest approach. This indicates that the errors of the Arrhenius-type equation exist in a more dispersed manner, while the random forest approach performs more reliably than the Arrhenius-type equation.

## 5. Conclusions

In this research, a random forest algorithm was used to model and predict the flow stress of the hot deformation of the 304 stainless steel alloy. The results showed that the random forest algorithm is efficient and performs more accurately than the traditional approach using the constitutive equation. The RMSE of the predicted stress by the random forest algorithm was smaller than the error obtained by the traditional equation. The errors by the random forest algorithm and the traditional equation were 4.93 and 6.93, respectively. The random forest algorithm was more efficient in developing the prediction model and in the computation. The relative percentage error study indicated that the random forest approach performs more reliably than the Arrhenius-type equation.

## Figures and Tables

**Table 1:** Coefficients of the polynomials.

Order	7	6	5	4	3	2	1	0
α	−276.12639	394.24715	−239.07069	79.83416	−15.87729	1.91202	−0.14018	0.01054
***n***	−2,000,302.2	2,567,176.7	−1,369,008.4	392,828.5	−65,565.3	6417.3	−353.8	15.7
***q***	−8$\times 10^{10}$	9.89$\times 10^{10}$	−5$\times 10^{10}$	1.34$\times 10^{10}$	−2$\times 10^{9}$	1.72$\times 10^{8}$	−7,600,110	527,533.9
***A***	−4.9$\times 10^{22}$	6.11$\times 10^{22}$	−3.1$\times 10^{22}$	8.4$\times 10^{21}$	−1.3$\times 10^{21}$	1.09$\times 10^{20}$	−4.8$\times 10^{18}$	8.96$\times 10^{16}$

**Table 2:** Errors of the material constants calculated by the polynomial functions. RMSE: root mean square error.

Constant	α	*n*	*q*	*A*
**RMSE**	$2.8255\times 10^{-6}$	0.007227	480.3273	$1.9854\times 10^{14}$

**Table 3:** Process parameters for random forest algorithm calculations.

Number of Trees	Regression Criterion	Depth of Tree	Minimum Samples to Split an Internal Node	Minimum Samples to Be at a Leaf Node	Input Attributes
600	Mean squared error	Unlimited	2	1	3

## Data Availability

Data are available from the corresponding author with the permission of W.-D. Choi.
